# Early results with the EyeCryl Phakic Toric intraocular lens implantation in keratoconus patients

**DOI:** 10.22336/rjo.2021.32

**Published:** 2021

**Authors:** Kepa Balparda, Claudia Marcela Vanegas-Ramírez, Tatiana Herrera-Chalarca, Laura Andrea Silva-Quintero

**Affiliations:** * Department of Research and Surgery, Black Mammoth Surgical, Medellín, Colombia; ** Department of Ophthalmology, Universidad Pontificia Bolivariana, Medellín, Colombia; *** Department of Clinical Research, Black Mammoth Surgical, Medellín, Colombia; **** School of Medicine, Universidad CES, Medellín, Colombia

**Keywords:** intraocular lens, keratoconus, refractive disorder, ophthalmology

## Abstract

**Objective:** Refractive management in keratoconus is challenging. Although some kinds of phakic intraocular lenses have been studied in keratoconus so far, no study evaluated the results of EyeCryl Phakic Toric intraocular lenses in this kind of patients.

**Materials and Methods:** This is a retrospective chart review study, including all keratoconus patients implanted with an EyeCryl Phakic Toric intraocular lens in at least one of their eyes by an experienced cornea surgeon in Colombia. Follow-up to 6 months after surgery was also included.

**Results:** A total of 20 eyes of 14 patients were included, with an average age of 29.3 ± 4.2 years. Spherical equivalent improved from a pre-surgical value of –10.31 D to +0.09 D at 6 months. 65% of the patients improved at least one line in the best-corrected distance visual acuity. At 6 months, 70% of the patients were within ± 0.50 D of spherical equivalent emmetropia. No complications occurred in any of the patients.

**Conclusion:** EyeCryl Phakic intraocular lenses are an excellent option in keratoconus patients with high refractive error.

**Abbreviations**:

KC = Keratoconus, P-IOL = phakic intraocular lenses, ICL = Implantable Collamer Lens, WTW = White to White, SD = standard deviation, ANOVA = an analysis of variance, UDVA = monocular uncorrected distance visual acuity, CDVA = corrected distance visual acuity

## Introduction

Keratoconus is the most common primary corneal ectasia worldwide and it is currently intensively researched. The visual dysfunction generated by this disease can cause severe effects on the patients’ quality of life and on public health systems worldwide. Although the use of glasses and contact lenses is common in patients with keratoconus, many of them desire to explore options that help reduce the refractive defects to more functional levels. The combination of an excimer laser with a corneal crosslinking (usually referred to as “Athens Protocol”) is a useful technique in patients with rather small refractive defects and with relatively good corneal conditions. Nevertheless, the management of high refractive defects in this kind of patients is a challenge.

The use of phakic intraocular lenses (P-IOL) has proven to be an effective and predictable technique in high myopic patients with normal corneas [**1**,**2**]. Some studies using Implantable Collamer Lens P-IOL (ICL; Staar Surgical; Monrovia, California, United States) have shown good predictability and effectiveness in patients with keratoconus [**3**]. EyeCryl Phakic Toric lenses (Biotech Vision Care; Ahmedabad, India) are a new posterior chamber P-IOL option, which have shown excellent results in normal corneas [**1**,**2**]. However, so far, no study that evaluates the behavior of EyeCryl Phakic Toric lenses in patients with keratoconus has been published.

The aim of this study was to present the short-term results (six-months follow-up) of patients with keratoconus, implanted with an EyeCryl Phakic Toric posterior chamber P-IOL, by a cornea surgeon experienced in the use of this type of technologies.

## Materials and methods

A retrospective study consisting of chart reviews, which aimed to evaluate the post-surgical results after six months of follow-up in patients with a confirmed diagnosis of keratoconus, who underwent EyeCryl Phakic Toric IOL implantation in at least one of their eyes.

*Pre- and post-surgical evaluation*

All patients included corresponded to private practice subjects of the first author of this manuscript and underwent a rigorous pre-surgical evaluation that included complete interrogation followed by a careful physical examination. The study adhered to the tenets of the Declaration of Helsinki.

Objective refraction and keratometry were obtained with a KR-800 autorefractometer (Topcon Corporation; Tokyo, Japan). Subsequently, subjective refraction without cycloplegia was obtained using a CV-5000S phoropter (Topcon Corporation; Tokyo, Japan), while measuring visual acuity with a PC-50S optotype monitor (Topcon Corporation; Tokyo, Japan). Then, a drop of 1% cyclopentolate (“Ciclopentolato Clorhidrato al 1%”, Oftalmoquímica Ltda, Cali, Colombia) was applied in each eye of the patients, every ten minutes, for a total of three doses. 45 minutes after the application of the first dose of cyclopentolate, a new objective and subjective refraction were measured with the same instruments, thus establishing refraction under pharmacological cycloplegia. 

Subsequently, the patient underwent a careful biomicroscopy through a SL-D2 slit lamp (Topcon Corporation, Tokyo, Japan), the intraocular pressure being taken by using a Goldmann type tonometer. A three-mirror lens was used to determine the aperture level of the chamber angle. The fundus was carefully evaluated by an indirect binocular ophthalmoscopy under dilation.

The ocular biometry was obtained by an IOLMaster 500 device (Carl Zeiss Meditec AG, Jena, Germany). A measurement of the pupillometry was also obtained under photopic and mesopic conditions by means of the Wavelight Allegro Topolyzer Vario (Alcon Inc, Forth Worth, United States). For the corneal tomography, an OCULUS Pentacam HR system (Oculus Optikgeräte GmbH, Wetzlar, Germany) was used. The lenses were calculated using Biotech Vision Care proprietary calculator available on their website. Given the non-availability of a digital caliper, the White to White (WTW) measurement was taken from the data drawn by the Pentacam, making an arbitrary adjustment reducing the value given by the equipment on 0.4 mm.

In the postoperative follow-up, it was the standard protocol of the first author of the present manuscript to evaluate patients on the first postoperative day, first week, first month, third month and sixth month. Data obtained in various postsurgical visits have been included. 

*Surgical Intervention*

All surgeries were performed under peribulbar anaesthesia by the main author with the same standardized technique as it follows: the patient was draped and the eye cleaned; then a 1.2 mm paracentesis and a 2.8 mm main incision were created, and the anterior chamber was filled with 2.4% Sodium Hyaluronate Ophthalmic Viscosurgical Device (Bio-Hyalur HV, Biotech Vision Care, Ahmedabad, India). The P-IOL was mounted and injected inside the anterior chamber. Afterwards, the lens was positioned in the correct toric markings, and all four haptics were positioned behind the iris over the ciliary sulcus. Then, the Ophthalmic Viscosurgical Device was removed, and 1% Acetylcholine was injected intracamerally to achieve proper pupillary miosis. Refractive target for all patients was +0.25.

*Statistical Analysis*

All qualitative values were expressed as absolute and relative proportions. All quantitative values were expressed as a mean with their corresponding standard deviation (SD).

For comparison of values between the different follow-up times, a one-way repeated measure analysis of variance (ANOVA) was performed. For all results, Wilks’ Lambda was expressed, along with an F and significance value. A significant value on ANOVA was always obtained, a post-hoc analysis with a Bonferroni correction being performed to detect which groups represented a statistically significant difference.

All tests were performed by using SPSS Statistics version 23 (International Business Machines Corporation, Armonk, New York, United Stated). The significance level for all the tests was set at a p value lower than 0.05.

## Results

A total of 20 eyes of 14 patients were included in this study. Nine (64.3%) patients were women, and the average age was 29.3 ± 4.2 years. Five (25.0%) eyes had a history of corneal collagen crosslinking, while two (10.0%) had a previous history of intracorneal ring segments implantation. Two (14.3%) patients had a prior Artiflex Toric IOL implantation in the contralateral eye.

**Fig. 1:**
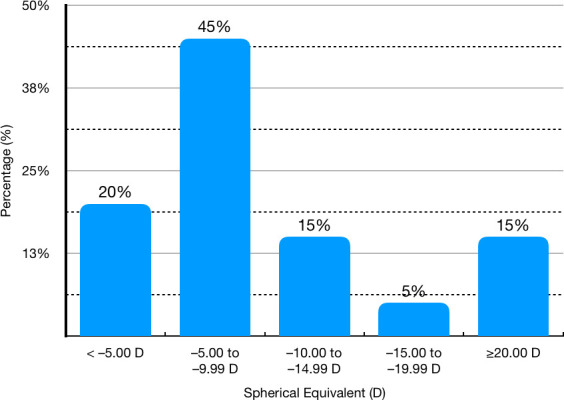
Distribution of preoperative spherical equivalent expressed in Diopters

Regarding Amsler-Krumeich classification, three (15.0%) eyes had a diagnosis of Grade 2 keratoconus, five (25.0%) eyes had a Grade 1, and the remainder had a forme fruste keratoconus. Preoperative characteristics and distribution of preoperative manifested SE are shown in **Table 1** and **Fig. 1**, respectively.

**Tabel 1:** Preoperative patient characteristics

	Mean ± SD	Minimum	Maximum
Sphere (D)	–8.40 ± 6.43	–0.75	–22.25
Cylinder (D)	–3.82 ± 1.62	–1.75	–7.75
SE (D)	–10.31 ± 6.18	–4.13	–23.63
IOP (mmHg)	11.45 ± 1.82	9	15
UDVA (LogMar)	1.58 ± 0.54	0.54	2.18
CDVA (LogMar)	0.15 ± 0.13	0.0	0.40
Cell density (Cells/mm3)	3054.63 ± 261.18	2678	3578
Corneal Astigmatism (D)	2.93 ± 1.46	1.40	6.90
Kmax (D)	47.16 ± 3.90	45.20	55.20
ACD (mm)	3.33 ± 0.32	2.90	4.00
SD = Standard Deviation, D = Diopter, SE = Spherical Equivalent, IOP = Intraocular Pressure, UDVA = Uncorrected Distance Visual Acuity, CDVA = Corrected Distance Visual Acuity, Kmax = Maximum Keratometry, ACD = Anterior Chamber Depth			

**Fig. 2:**
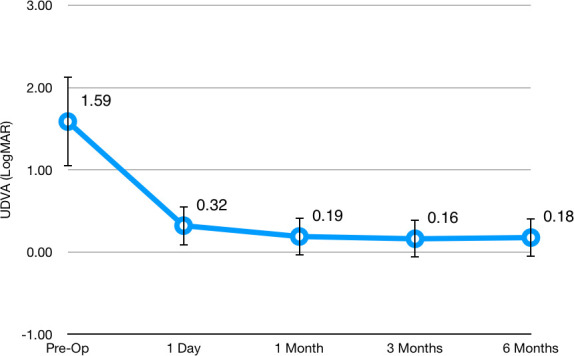
Distribution of Uncorrected Distance Visual Acuity (UDVA) during follow-up, expressed in LogMAR. Circles represent the mean and the whiskers represent the standard deviation of the mean. One-way repeated measures ANOVA Wilks’ Lambda = 0.130, F(4, 16) = 26.876, p < 0.01. Statistically significant difference was observed from preoperative to all other groups (Bonferroni p < 0.01 for all comparisons). All other comparisons proved non-significant (Bonferroni p > 0.05)

**Fig. 3:**
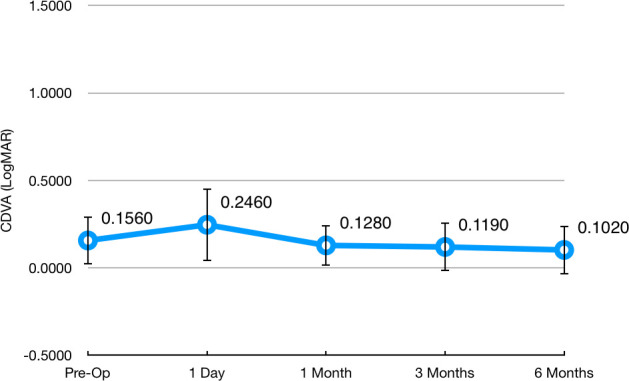
Distribution of Corrected Distance Visual Acuity (CDVA) during follow-up, expressed in LogMAR. Circles represent the mean and the whiskers represent the standard deviation of the mean. One-way repeated measures ANOVA Wilks’ Lambda = 0.405, F(4, 16) = 5.873, p < 0.01. Statistically significant difference was observed when comparing preoperative to 6-months visit (Bonferroni p < 0.01). All other comparisons proved non-significant (Bonferroni p > 0.05)

**Fig. 4:**
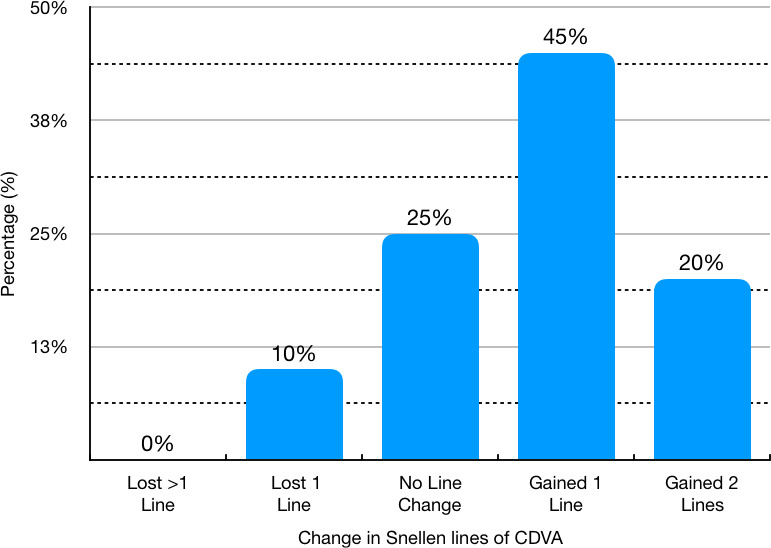
Distribution of changes in Corrected Distance Visual Acuity (CDVA) when comparing 6-months values to preoperative values

Initial monocular uncorrected distance visual acuity (UDVA) was 1.58 ± 0.54 LogMAR, and it improved to 0.17 ± 0.23 LogMAR 6 months after surgery. **Fig. 2** and **3** show the evolution of UDVA and corrected distance visual acuity (CDVA) during the follow-up course, respectively. The safety index (ratio of postoperative CDVA to preoperative CDVA) was 1.52 at 6 months after surgery. Only one eye (5.0%) lost one line of CDVA at 6 months after surgery. No patient lost more than one line of CDVA. **Fig. 4** shows the distribution of CDVA lost/ gained for the patients in the study when comparing preoperative values to 6-months ones.

**Fig. 5:**
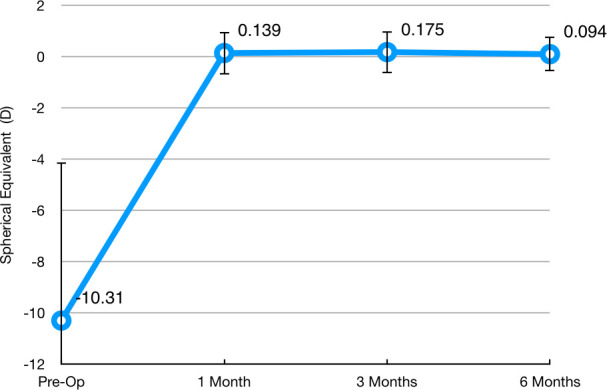
Distribution of Spherical Equivalent during follow-up, expressed in Diopters. Circles represent the mean and the whiskers represent the standard deviation of the mean. One-way repeated measures ANOVA Wilks’ Lambda = 0.169, F (3, 17) = 27.951, p < 0.01. Statistically significant difference was observed from preoperative visit to all other groups (Bonferroni p <0.01 for all comparisons). All other comparisons proved non-significant (Bonferroni p > 0.05)

Manifest sphere decreased from a preoperative value of –8.40 ± 1.43 D to +0.42 ± 0.12 D at 6 months after surgery (paired samples student-t p<0.01). Manifest cylinder also decreased from a preoperative value of –3.82 ± 0.36 D to –0.66 ± 0.18 (paired samples student-t p< 0.001). **Fig. 5** shows the evolution of SE before surgery and during the follow-up period.

When comparing intraocular pressure values, one-way repeated ANOVA showed a significant difference (Wilks’ Lambda = 0.309, F(4,16) = 8.958, p < 0.01) between the groups. On post-hoc evaluation, only intraocular pressure at 1 day after surgery proved to be different from the rest (Bonferroni p < 0.01). The rest of the groups (preoperative, 1 month, 3 months and 6 months) proved to be equal (Bonferroni p = 1.0). Intraocular pressure before surgery was 11.45 ± 1.82 mmHg and on first day was 14.80 ± 2.89 mmHg.

**Fig. 6:**
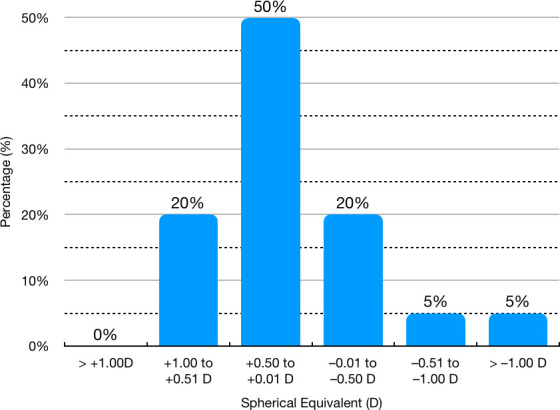
Distribution of Spherical Equivalent at 6 months after surgery, expressed in Diopters

At 6 months, 14 (70%) eyes were within ± 0.50 D of spherical equivalence emmetropia. **Fig. 6** shows the distribution of spherical equivalence in the study group at 6 months after surgery. Endothelial cell density was 3054.63 ± 261 cells/ mm2 before surgery and it decreased to 3011.27 ± 279 cells/ mm2 at 6 months (mean change 43.36 ± 47.03 cells/ mm2, p = 0.01). Mean endothelial cell loss at 6 months was small (≈1.41%).

One (5%) eye had ocular hypertension (24 mmHg) the first day after surgery. It resolved in 48 hours without the need for anti-glaucoma drops. No cases of sustained increase in intraocular pressure or glaucoma were diagnosed. No patient developed cataract or any other complications.

## Discussion

The refractive management of patients with keratoconus is a complex task that requires extensive experience in these kinds of conditions. Although some authors have proposed the use of an excimer laser combined with a Corneal Crosslinking (“Athens Protocol”, “Crete Protocol”, among others), these types of approaches are not risk-free, and could potentially generate a greater corneal alteration in patients with structural abnormality in the anterior ocular segment.

The use of P-IOL represents an attractive option in this kind of patients, as it offers the possibility of making corrections of very high refractive defects, without structurally weakening the cornea. In addition, the P-IOL implant has been shown not to increase higher-order aberrations [**3**,**4**], one of the main reasons of vision loss in many ectatic patients. Therefore, they could theoretically represent a much safer option in patients with sub-optimal corneas, including those with keratoconus.

So far, several studies have shown that the implant of ICL P-IOL is effective and safe in patients with corneal ectasia. Emerah and collaborators [**3**] evaluated a total of 14 eyes with keratoconus implanted with toric ICL lenses, finding a significant improvement in uncorrected vision, and with 42.8% of patients gaining one line in their vision with correction. Follow-up of up to three years demonstrated the stability of these lenses in patients with ectasia. For example, Kamiya and collaborators [**5**] published their follow-up in 21 eyes implanted with toric ICL lenses, finding that after 3 years, 67% and 86% of patients continued with manifest refractions ± 0.5 D and ± 1.0 D of the planned, respectively. There were no statistically significant changes in refraction or keratometry during follow-up.

EyeCryl Phakic Toric IOLs, produced by Biotech Vision Care, are a kind of phakic posterior chamber lens alternative to ICL, at a significantly more “economically viable” [**6**] price than the latter. Their effectiveness and safety have been demonstrated in highly-myopic patients with normal corneas [**1**,**2**]. Nonetheless, so far, no published study has previously demonstrated whether this kind of P-IOLs were useful in patients with corneal ectasia.

In the study presented, the authors evaluated a total of 20 eyes, following them for a total of six months after the intervention. These were patients with relatively high refractive defects, as evidenced by the average spherical equivalent of –10.31 D, with extreme values, such as a patient having –23.63 D in its spherical equivalent. The follow-up of the patients showed a great improvement in the uncorrected visual acuity that was sustained through the different months of follow-up. Subjective refraction also showed a great improvement, changing from a preoperative value of –8.40 ± 1.43 D to a postoperative value of +0.42 ± 0.12 D after six months of follow-up.

Probably the most important thing in the follow-up was the improvement in the visual potential of the patients, demonstrated by the fact that 65% of the individuals improved at least one line in the best corrected vision, a value comparable to what has been previously reported with other brands of P-IOL in patients with keratoconus [**3**]. It has been postulated that this improvement in corrected vision may be secondary to the artefactual minimization that occurs with highly-myopic glasses, an optical phenomenon that does not happen with intraocular lenses.

The present study also demonstrated the safety of this type of interventions, owing any complication that was not demonstrated beyond an isolated episode of ocular hypertension during the first postoperative day that quickly improved. None of the patients showed sustained elevations in intraocular pressure, cataract, retinal detachment, or any other type of alterations attributable to the lens implant.

As suggested above, one of the most important elements that guarantees the success in the implantation of P-IOL in patients with keratoconus lies in the adequate selection of patients. It is the personal protocol of the main author of the present study (K. B.) to only consider the implantation of P-IOL in patients who achieved a visual acuity with the phoropter equal to or greater than 20/ 50. The above is justified in the fact that, being in the intraocular space, P-IOL does not generate any improvement in corneal aberrations, so the vision after their implantation will be much more similar to that obtained with glasses than the one obtained with contact lenses. Patients with higher levels of high-order aberrations may not be good candidates for P-IOL implantation and would have a better outcome with other types of approaches. In other words, P-IOLs will have a better outcome in those patients whose visual complaints are due to a high uncorrected refractive, than corneal deformity itself. 

It is undeniable that there are some limitations to the present study. It is a relatively short follow-up (6 months) period. Longer follow-ups would be recommended to determine refractive behavior over time, and to evaluate the occurrence of other complications, such as cataracts and corneal endothelial depletion. Additionally, the number of subjects studied is relatively small, which could have affected the study’s power to find differences. However, the changes between the pre-surgical and post-surgical state have been large enough to be detected within the small population.

## Conclusions

The refractive management of patients with keratoconus is complex, and there is a significant number of surgical techniques to improve the condition of patients. The implant of EyeCryl Phakic Toric lenses is a safe, effective, and reliable technique in patients with keratoconus, achieving improvement in visual potential in more than half of them. The success of the intervention will depend, to a large extent, on the proper selection of patients, reserving this type of interventions for those who do not show high levels of high-order aberrations and have a good vision that can be corrected with the phoropter.

**Conflict of Interest statement**

Authors state no conflict of interest.

**Informed Consent and Human and Animal Rights statement**

Informed consent has been obtained from all individuals included in this study.

**Authorization for the use of human subjects**

Ethical approval: The research related to human use complies with all the relevant national regulations, institutional policies, is in accordance with the tenets of the Helsinki Declaration, and has been approved by the institutional review board of Black Mammoth Surgical, Medellín, Colombia.

**Acknowledgements**

None.

**Sources of Funding**

No sources of support in the form of grants, equipment, drugs, etc.

**Disclosures**

The first author (K. B.) is a paid speaker and researcher for EyeCryl Phakic intraocular lenses (Biotech Vision Care, Ahmedabad, India). He did not receive any kind of compensation or payment for the management of the patients in this study nor for the publication of this article. Biotech Vision Care had no role in the design, data collection, data analysis, or manuscript writing for this study. The other authors have no conflicts of interest to disclose.
